# Peanut Shell Powder as a Sustainable Modifier and Its Influence on Self-Healing Properties of Asphalt

**DOI:** 10.3390/ma16206618

**Published:** 2023-10-10

**Authors:** Bo Wang, Junan Shen, Shuang Li, Wei Wang

**Affiliations:** 1 Zhongyifeng Construction Group Co., Ltd., Suzhou 215131, China; sum77560702@outlook.com; 2 Jiangsu Technology Industrialization and Research Center of Ecological Road Engineering, Suzhou University of Science and Technology, Suzhou 215011, China; ls3215911489@163.com; 3 Department of Civil Engineering and Construction, Georgia Southern University, Statesboro, GA 30458, USA; jshen@georgiasouthern.edu; 4 School of Civil Engineering, Chongqing Jiaotong University, Chongqing 400074, China

**Keywords:** peanut shell powder, asphalt, self-healing, sustainable

## Abstract

This paper investigated, for the first time, the feasibility of using peanut shell powder, a plant waste residue, as a modifier for asphalt, particularly its self-healing ability. Modified asphalt samples were prepared using varying particle size ranges and concentrations of peanut shell powder. Various tests, including fatigue–healing–fatigue tests, high- and low-temperature rheological property tests, penetration tests for conventional performance, and atomic force microscopy scans, were conducted to investigate the effects of peanut shell powder on the self-healing performance and other properties of asphalt. The results showed that the porous structure of peanut shell powder was able to absorb light components within the asphalt and release them under load, thus improving the self-healing and fatigue resistance properties of the modified asphalt. Experimental conditions such as temperature, healing time, and fatigue damage level also influenced the self-healing performance of asphalt. Additionally, peanut shell powder could increase the dynamic viscosity and high-temperature rheological property of modified asphalt while reducing its temperature susceptibility. However, it had a negative impact on the low-temperature ductility and creep rate, which could potentially lead to premature cracking of asphalt pavement in colder regions. Increasing the content of peanut shell powder and reducing its particle size within a certain range had positive effects. When the content of peanut shell powder was 4% and the particle size range was 80–100 mesh, the overall performance of modified asphalt was satisfactory.

## 1. Introduction

Asphalt mixtures are widely used in modern road engineering. The interaction between environmental factors and applied loads can induce micro-cracks within the asphalt matrix, leading to progressive cracking of the asphalt pavement [1,2]. This leads to a reduction in the service life of the road engineering, resulting in increased maintenance and preservation costs. Additionally, frequent maintenance measures necessitate more operation of hot mix asphalt blends and equipment, exacerbating greenhouse gas emissions [3]. Asphalt, as a viscoelastic material, possesses self-healing properties, whereby minor damages and micro-cracks in the asphalt pavement have the ability to undergo self-repair to a certain extent. However, this self-healing process is time-consuming, which makes interrupting traffic for extended periods impractical [4]. Therefore, since the 1960s, scholars have been devoted to researching methods to enhance the self-healing performance of asphalt [5,6,7,8].

In the past several decades, there has been an abundance of laboratory research findings that have demonstrated significant advancements in enhancing the self-healing performance of asphalt. These findings primarily fall into three categories: the addition of self-healing agents, induction heating, and microwave heating methods [9,10,11]. The rejuvenators in self-healing agents are typically derived from light oils, such as waste engine oil or waste vegetable oil, which are commonly used to restore the light components in asphalt. These agents are formulated using carriers such as microcapsules, hollow fibers, or capillary fibers [12,13]. The basic principle of improving the self-healing performance of asphalt using self-healing agents lies in the fact that when asphalt undergoes external influences and develops micro-cracks, it also leads to the rupture of dispersed self-healing agents within the asphalt, releasing rejuvenator. The rejuvenator can fill the micro-cracks in the asphalt, thereby achieving the restoration of asphalt performance [14,15]. In order to ensure that the self-healing agents in asphalt mixtures are not damaged during the mixing process and possess sufficient high-temperature resistance, chemical agents and high-molecular-weight polymers are commonly used as the shells (carriers) of the self-healing agents [16,17]. This undoubtedly increases production costs and may have adverse environmental effects [16]. Another reason why these healing agents are difficult to be implemented in production is that their dispersion in asphalt needs to be uniform, which requires complex preparation processes, considering their size is typically in the micrometer range [18]. Compared to the method of adding healing agents, the heating method does not require the addition of any rejuvenators. The induction heating method is based on electromagnetic induction and Joule heating principles, where heat is generated in a material when an electric current passes through it [19]. In induction heating, the material is placed in an artificially induced magnetic field, which generates eddy currents that heat the material. Asphalt itself is not a conductive material; therefore, researchers have achieved conductivity in asphalt by incorporating conductive fibers or fillers such as steel fibers, graphite, etc., into the asphalt [20]. When the temperature of the conductive material increases, it also heats the asphalt, increasing its fluidity and facilitating the automatic healing of micro-cracks. This method necessitates the use of substantial induction heating devices, which can be cumbersome in practical pavement engineering applications. Another heating method is microwave heating, which involves adding dielectric materials such as steel wool and iron oxides to the asphalt. These materials generate heat under microwave radiation, thereby heating the asphalt and enhancing its self-healing capability [21,22,23]. However, according to the World Health Organization, microwave energy can be absorbed by the human body and result in thermal injuries. Furthermore, microwaves are typically subject to planar reflections, and controlling these reflections is challenging [24]. This also limits the application of microwave heating in asphalt pavement engineering.

In recent years, some nanometer materials such as nanoclays, nano-silica, and nano-titanium dioxide have also been used to improve the self-healing performance of asphalt [4]. These tiny particles have characteristics such as small particle size distribution and high surface area, which can accelerate the flowability of asphalt. They can also adsorb the light component (saturated and aromatic hydrocarbons) in the asphalt and release them under load, thereby enhancing the self-healing performance of asphalt [11,25,26,27]. These research findings provide new insights for enhancing the self-healing performance of asphalt using other particulate materials.

The asphalt self-healing agents mentioned above are limited by drawbacks such as complex manufacturing processes, high costs, and inconvenient application, which have hindered their widespread use in practical engineering projects. Exploring a sustainable new material that is readily available, easy to process, and cost-effective has become a bottleneck issue in this field. Studies have shown that the addition of plant powder to asphalt can significantly improve its high-temperature performance, fatigue resistance, and aging resistance, which is attributed to its porous structure [25,28]. The light fractions in asphalt have the lowest stiffness and are the most susceptible to aging [29]. Plant powder with a high porosity structure can absorb certain light components and gradually release them under external environmental influences. This principle opens up possibilities for using plant powder to enhance the self-healing performance of asphalt. Peanut shell powder is a readily available plant waste, with an annual production in China exceeding tens of millions of tons. Researchers have used peanut shell powder as a modifier for asphalt and evaluated the performance of the modified asphalt [30]. The results indicate that the porous structure of peanut shell powder can suppress the carbon emissions of asphalt after heating. However, there are currently no reports on the impact of peanut shell powder on the self-healing performance of asphalt.

The aim of this study was to investigate the influence of peanut shell powder on the performance of asphalt, particularly its effect on self-healing performance. Two different source base asphalts and four different particle sizes of peanut shell powder were selected. Modified asphalts with varying mass ratios of peanut shell powder to asphalt were prepared. The self-healing performance and fatigue resistance of the different modified asphalts were tested and analyzed using a dynamic shear rheometer. Additionally, the penetration, softening point, ductility, viscosity, and rheological properties of the modified asphalts were compared. Finally, atomic force microscopy was employed to analyze the microstructural characteristics of the samples.

## 2. Materials and Methods

### 2.1. Materials

**Figure 1:**
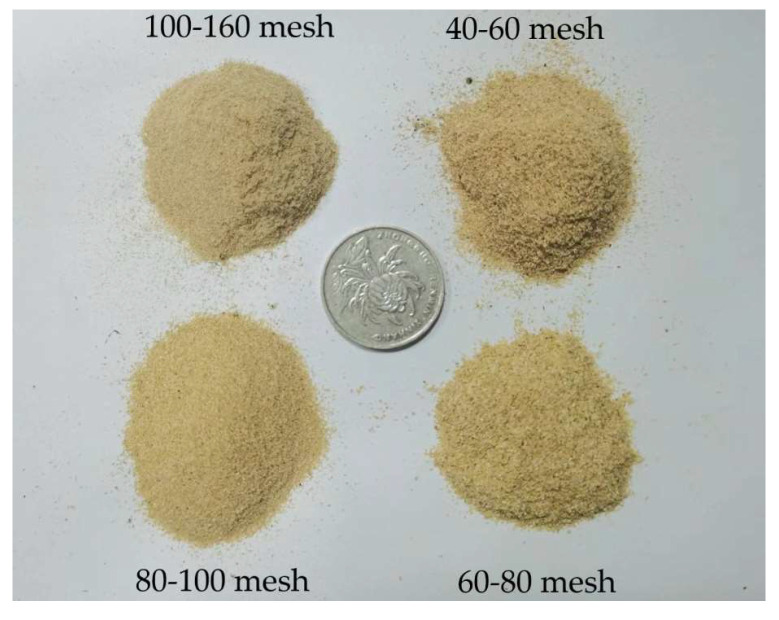
The peanut shell powder.

This investigation selected four different particle size ranges of peanut shell powder (40–60 mesh, 60–80 mesh, 80–100 mesh, 100–160 mesh) from Yancheng City, Jiangsu Province, China, as shown in Figure 1. The particle size range of peanut shell powder spans from 40 to 160 mesh, corresponding to approximately 90 to 380 μm. In comparison, the particle size range for commonly utilized asphalt self-healing agents generally falls within 60 to 200 μm. To broaden the scope of our examination, we’ve chosen this specific particle size range for peanut shell powder, which is also commonly encountered during its processing. The basic properties are shown in Table 1. The Shuanglong base asphalt was sourced from South Korea, while the Shell base asphalt came from Singapore, and their basic properties are shown in Table 2.

### 2.2. Methods

#### 2.2.1. Preparation of Modified Asphalt with Peanut Shell Powder

Based on our preliminary research findings, in order to mitigate the aging effect of high temperatures on asphalt and simultaneously ensure good workability, the base asphalt was heated to 130 °C. Peanut shell powder was then added to the base asphalt in mass ratios of 0%, 1%, 2%, 3%, 4%, 5%, and 6%. The mixture was stirred evenly using a glass rod. Subsequently, a high-speed shear dispersion emulsifier was used to shear the peanut shell powder-modified asphalt. The shear speed was set at 2000 rad/min, the shearing time was 0.5 h, and the stirring temperature was 150 °C. Furthermore, in order to control variables, the base asphalt was also subjected to the same shearing conditions. Furthermore, to prevent errors caused by segregation, all test samples were remixed prior to the experiments, and they were prepared at the same temperature of 130 °C. Due to the multiple variables, the modified asphalt was labeled according to the following principles: The base asphalt was identified as Shuanglong asphalt, peanut shell powder with a particle size range of 40–60 mesh and a content of 2% was labeled as SL-40-2. Other samples were labeled following the same principle, as shown in Table 3.

#### 2.2.2. Fatigue–Healing–Fatigue Test for Peanut Shell Powder-Modified Asphalts Based on Dynamic Mechanical Analysis

Dynamic Mechanical Analysis (DMA) is a crucial technique for measuring the viscoelastic dynamic changes in asphalt within a specific temperature and loading frequency range. In comparison to other methods, DMA boasts advantages such as high precision and strong reproducibility [31]. In DMA, sinusoidal alternating stress is applied to asphalt specimens using a dynamic shear rheometer (DSR), and the changes in strain are measured. For linear viscoelasticity, both stress and strain exhibit sinusoidal variations, with strain lagging behind stress. The resulting strain *ε*, stress *σ*, and phase angle *δ* during each loading cycle can be obtained, and the complex shear modulus G* can be calculated according to Equation (1) [32].

(1) $$G^{*}=\frac{\sigma }{\varepsilon }$$

**Figure 2:**
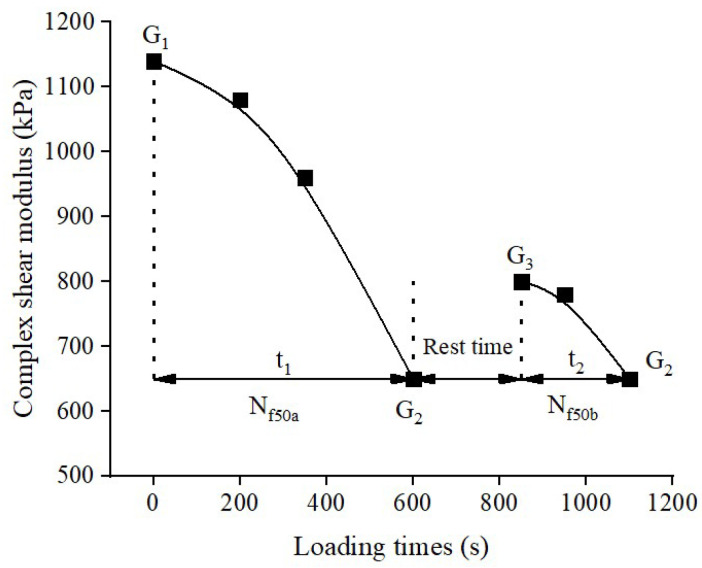
Schematic diagram of Fatigue–Rest–Fatigue test.

Figure 2 shows how the change in the complex shear modulus G* during the fatigue–healing–fatigue test can be used to evaluate the asphalt’s self-healing performance [33]. Firstly, fatigue damage test is conducted on the asphalt using a DSR. The test is stopped when the complex shear modulus decreases to 50% (or 15% or 30%) of the initial modulus *G*_1_, and the modulus at this point is denoted as *G*_2_. Then, under given temperature value, the loading is stopped and crack repair is performed during a resting period called the healing time (rest time). Finally, the second fatigue damage test is conducted, and the initial modulus during this stage is denoted as *G*_3_. The test is stopped when the modulus value reaches the same level as *G*_2_. The self-healing capacity of the asphalt specimen can be calculated using Equation (2), and the calculated value is referred to as the healing index HI. A higher healing index indicates better self-healing capacity of the asphalt [34]. The experimental parameters used in this study were as follows: Dynamic Shear Rheometer (DSR) operated in time sweep mode with a frequency of 10 Hz, controlled shear strain of 10%, parallel plate diameter of 8 mm, and a gap distance of 2 mm. The test temperatures were 20, 25, and 30 °C. The healing times were 10, 20, and 30 min, and the degrees of damage were set at 15%, 30%, and 50%. Each test was conducted in at least three parallel experiments, and the average value of the measurements with a coefficient of variation less than 15% was taken as the final result.

(2) $$HI=\frac{G_{3}-G_{2}}{G_{1}-G_{2}}\;\;\;\;\;$$

where *G*_1_ represents the initial complex shear modulus during fatigue damage testing, kPa; *G*_2_ represents the complex shear modulus at a specified level of modulus reduction, kPa; *G*_3_ represents the initial complex shear modulus during the second fatigue damage testing after a certain healing time, kPa.

#### 2.2.3. The Fatigue Resistance of Peanut Shell Powder-Modified Asphalts

This study evaluated the fatigue resistance of peanut shell powder-modified asphalt using the *N_f_*_50_ method and fatigue factor [35]. The testing procedure for the *N_f_*_50_ method was the same as described in Section 2.2.2, where the loading time *N_f_*_50a_ (t_1_) was measured when the asphalt sample’s initial shear modulus decreased by 50%, and the loading time *N_f_*_50b_ (t_2_) was measured when the modulus decreased to 50% of the initial shear modulus after healing. The fatigue life *N_f_*_50_ was calculated using Equation (3). The test parameters were as follows: test temperature of 25 °C, healing time of 10 min, fatigue damage degree of 50%, and controlled strain of 10%. The fatigue factor was primarily based on the rheological properties of the asphalt. The testing method for the fatigue factor was similar to that of the *N_f_*_50_ method, with the difference being that the DSR test used a single frequency sweep mode with a controlled shear strain of 4%, while keeping other test conditions constant. The complex shear modulus G* and phase angle *δ* were recorded during the first loading, and the fatigue factor G*·*sinδ* of the modified asphalt was calculated using Equation (4).

(3) $$\;\;\;N_{f50}=N_{f50a}+N_{f50b}$$

(4) $$\;\;G^{*}*sin\delta =G^{*}\times sin\delta$$

#### 2.2.4. Conventional Tests of Peanut Shell Powder-Modified Asphalts

The properties of the modified asphalt, including 25 °C penetration, softening point at 10 °C, ductility at 10 °C, and dynamic viscosity at 60 °C, were measured using SYD-2801E automatic penetration tester, SYD-2806F digital display softening point tester, LYY-10A-1 ductility tester, and WSY-08 dynamic viscosity tester. The equipment was sourced from Xinnuo Instrument Equipment Co., Ltd., Shanghai, China. At least three samples were tested for each experiment, and the average value was recorded as the final result. At least three samples were tested for each experiment, and if the coefficient of variation did not meet the specification [36], the experiment was repeated, and the average value that met the requirements was recorded as the final result.

#### 2.2.5. The Rheological Properties of Peanut Shell Powder-Modified Asphalts

The rheological characteristics of peanut shell powder-modified asphalt at high temperatures were investigated using Dynamic Shear Rheometer (DSR). The experimental equipment used was the MALVERN CvO100 Dynamic Shear Rheometer from the Malvern City, UK. The controlled strain in the tests was set at 10%, with a parallel plate diameter of 25 mm and a gap distance of 1 mm. The test temperature was 64 °C, and the frequency was set at 10 Hz. The experiments were repeated three times. The main viscoelastic parameters of the modified asphalt, including complex modulus (G*) and phase angle (*δ*), were obtained in this study. Based on G* and *δ*, the rutting resistance factor G*/*sinδ* was calculated and used as an evaluation index for the high-temperature performance of the modified asphalt [37].

The low-temperature rheological properties of peanut shell powder-modified asphalt at −12 °C were studied using the Bending Beam Rheometer (BBR) test. The equipment used for this study was the CANNON TE-BBR Bending Beam Rheometer from the Cleveland, OH, USA. The beam-shaped specimens had dimensions of 127 × 12.70 × 6.25 mm, and the tests were repeated three times. All samples were soaked in an ethanol bath for 1 h and subjected to a constant force of 980 ± 50 mN for 240 s before testing. After the test, the stiffness (S) and creep rate (m-value) were obtained using a 60 s mid-point deflection. The S-value represents the deformation capacity of the modified asphalt, while the m-value represents the stress relaxation capacity. A smaller S-value and a larger m-value indicate better low-temperature cracking resistance of the modified asphalt.

#### 2.2.6. Atomic Force Microscope (AFM)

**Figure 3:**
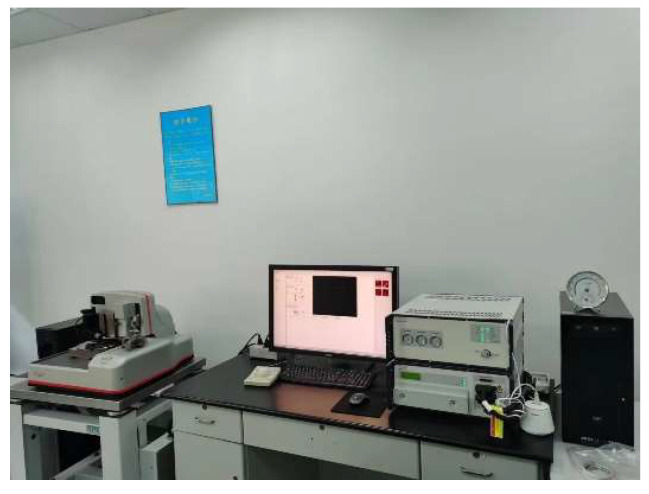
Atomic force microscopy.

The nanoscale morphology of the peanut shell powder-modified asphalt was scanned and tested using the Dimension Icon atomic force microscope (AFM) produced by Bruker in Karlsruhe, Germany, as shown in Figure 3. The scanning mode used was PeakForce-QNM, with a scanning probe of 0.4 N/µm silicon nitride. The sample was prepared using the drop-casting method, and the scanning range was 20 μm × 20 μm. In addition to the microscopic morphology images, AFM testing can also provide micro-characteristic parameters of the asphalt, namely, root mean square of height (R_q_), maximum and minimum height difference (R_max_), and Young’s modulus (DMT). R_q_ and R_max_ are commonly used to characterize the roughness and smoothness of asphalt micro-interfaces, where a lower value indicates a flatter micro-interface. The DMT modulus represents the elastic modulus of asphalt at the microscopic scale, and a higher value indicates a greater resistance to elastic deformation [1].

## 3. Results and Discussion

### 3.1. Self-Healing Properties of Peanut Shell Powder-Modified Asphalt

#### 3.1.1. Effect of the Peanut Shell Powder Content on the HI of Modified Asphalt

**Figure 4:**
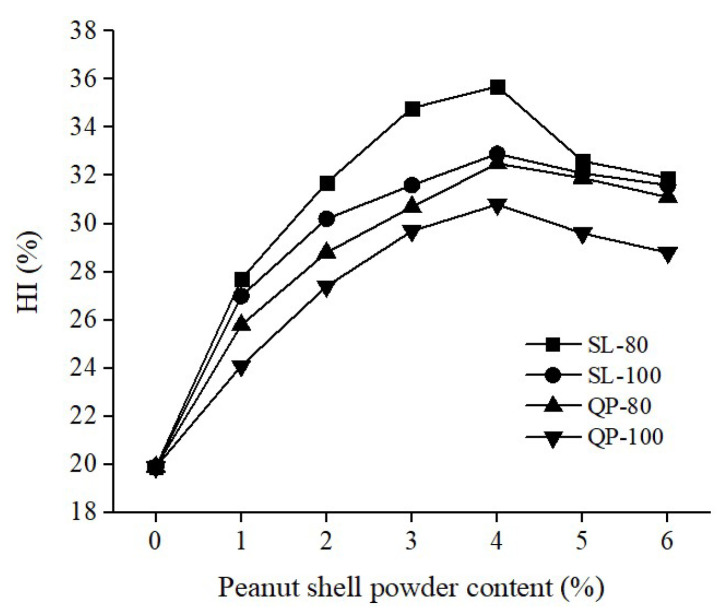
Effect of peanut shell powder content on the HI of modified asphalt.

The test results are shown in Figure 4. The fatigue damage testing was conducted at a temperature of 25 °C with a healing time of 10 min, a fatigue damage degree of 50%, and a controlled strain of 10%. The effect of different peanut shell powder contents (0%, 1%, 2%, 3%, 4%, 5%, and 6%) on the self-healing performance of modified asphalt was investigated, using two different particle size ranges of peanut shell powder (80–100 mesh and 100–160 mesh). With the increase in the addition of peanut shell powder, the HI of the modified asphalt initially showed an upward trend followed by a downward trend. When the base asphalt was Shuanglong, the peanut shell powder had a particle size range of 80–100 mesh, and the addition was 4%; the HI was 35.7%, showing the highest increase, with a 79.4% improvement compared to the base asphalt. This indicates that peanut shell powder enhances the self-healing performance of asphalt. When the addition of peanut shell powder is low, the absorption of light components in asphalt is limited, resulting in limited repair of fatigue damage. At lower concentrations of peanut shell powder, there is limited absorption of the asphalt’s light components, causing restricted damage repair. Conversely, at high concentrations, the increased viscosity impedes the asphalt molecule mobility, thereby reducing healing efficiency. Additionally, the HI of the peanut shell powder-modified Shuanglong asphalt was higher than that of the Shell asphalt, which may be related to the differences in the compositional properties of the two asphalts.

#### 3.1.2. Effect of the Particle Size of Peanut Shell Powder on the HI of Modified Asphalt

**Figure 5:**
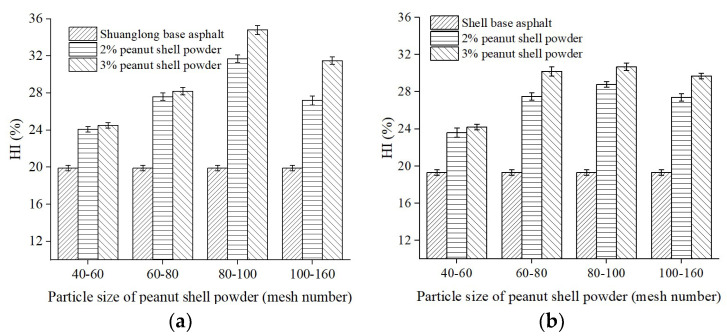
Effect of particle size of peanut shell powder on the HI of modified asphalt. (**a**) Modified Shuanglong asphalt; (**b**) Modified Shell asphalt.

The testing process was the same as in Section 3.1.1. Two different contents of peanut shell powder (2% and 3%) were selected, and the test results are shown in Figure 5. Under the same peanut shell powder content conditions, the HI of the modified asphalt showed an initial increase and then a decrease trend with a decrease in particle size of the peanut shell powder. When the particle size of the peanut shell powder was 80–100 mesh, the HI of the modified asphalt using two different base asphalt, Shuanglong and Shell, reached its maximum values, which were 34.8% and 30.7%, respectively. This could be attributed to the fact that as the particle size of peanut shell powder decreases, its specific surface area increases, leading to a stronger ability to absorb light oil components in the asphalt. However, when the particle size range of peanut shell powder exceeds 100 mesh, the proportion of internal cavity volume in the particles decreases, resulting in a weakened capacity to store and retain light oil components. Additionally, under consistent experimental conditions, the self-healing index of the modified asphalt using Shuanglong asphalt as the base asphalt remained greater than that of Shell asphalt.

#### 3.1.3. Effect of the Test Temperature on the HI of Modified Asphalt

**Figure 6:**
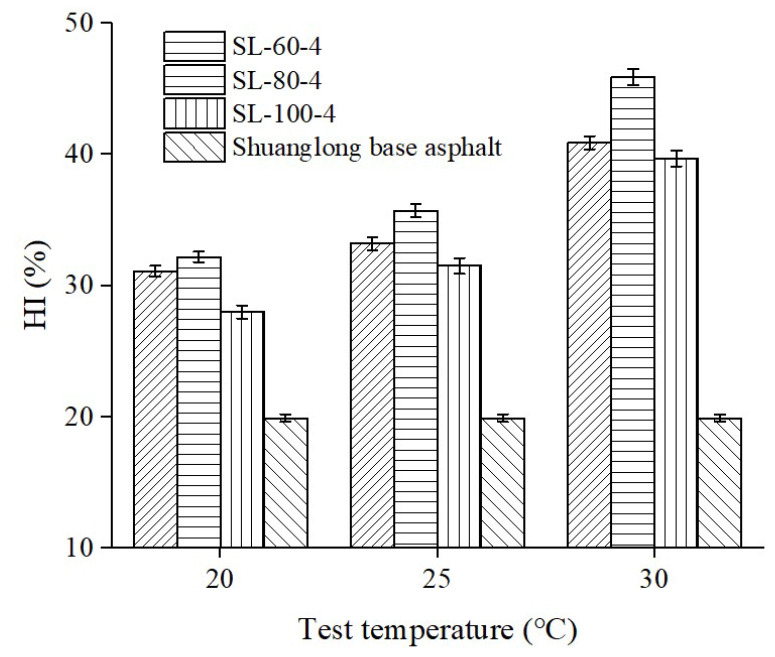
Effect of test temperature on the HI of modified asphalt.

To investigate the effect of test temperature on the HI of peanut shell powder-modified asphalt, the variation trend of the healing index was tested for peanut shell powder-modified asphalt at test temperatures of 20 °C, 25 °C, and 30 °C. Based on previous results, the optimal content of peanut shell powder (4%) and Shuanglong base asphalt were selected to prepare the modified asphalt. The particle size ranges of the peanut shell powder were 60–80 mesh, 80–100 mesh, and 100–160 mesh, respectively. The test results are shown in Figure 6. The HI of all asphalt samples increased with the increase in test temperature, which is consistent with previous research findings [4,32]. The maximum HI value of 45.9% was achieved at a test temperature of 30 °C and a particle size range of 80–100 mesh for peanut shell powder. Asphalt is viscoelastic, so as temperature rises, the increased molecular mobility allows for better filling of microcracks, enhancing self-healing [10]. In addition, regardless of the test temperature, the modified asphalt with a particle size range of 80–100 mesh for peanut shell powder exhibited the highest HI, which is consistent with the experimental results from Section 3.1.2.

#### 3.1.4. Effect of the Healing Time on the HI of Modified Asphalt

**Figure 7:**
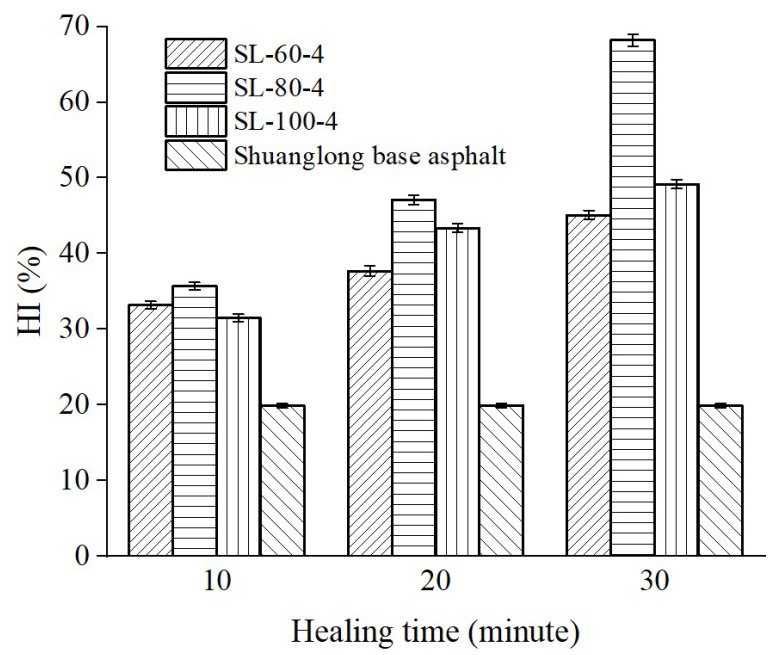
Effect of healing time on the HI of modified asphalt.

To investigate the effect of healing time on the HI of peanut shell powder-modified asphalt in the fatigue–healing–fatigue test, the variations in HI were tested for peanut shell powder-modified asphalt at healing times of 10 min, 20 min, and 30 min. The content and particle size range of peanut shell powder were the same as in Section 3.1.3. The test temperature was 25 °C, which is typically the temperature at which fatigue failure of asphalt materials occurs [35]. The test results are shown in Figure 7. The HI of all asphalt samples increased with prolonged healing time. The maximum value was achieved when the healing time was 30 min and the particle size of peanut shell powder was 80–100 mesh, reaching 68.2%. According to molecular motion theory, asphalt molecules continuously move randomly. Longer healing times allow oils released by the peanut shell powder to address more damage areas, thus improving self-healing [34]. To save time during the experimental process, 10 min was chosen as the healing time for other experiments.

#### 3.1.5. Effect of the Damage Degree on the HI of Modified Asphalt

**Figure 8:**
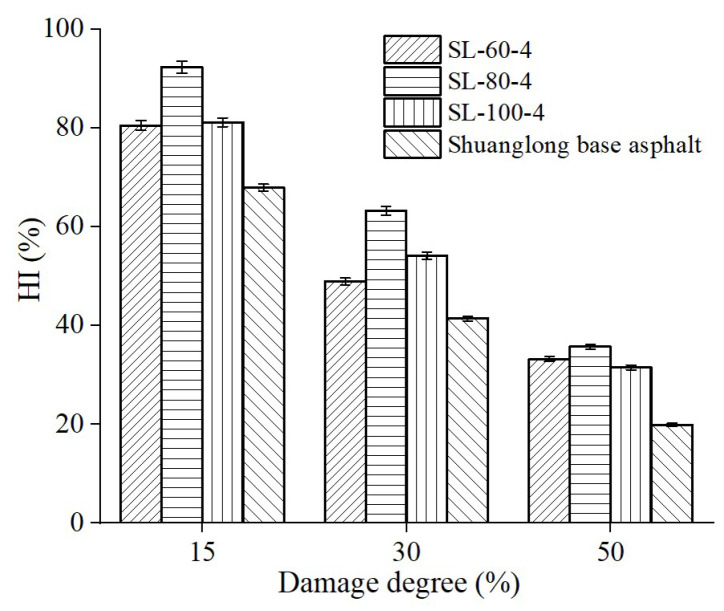
Effect of damage degree on the HI of modified asphalt.

The damage degree is defined as the percentage of the complex shear modulus (*G*_2_) after the first fatigue loss test compared to the initial complex shear modulus (*G*_1_), i.e., *G*_2_/*G*_1_, as described in Section 2.2.2. The variation trend of the HI for peanut shell powder-modified asphalt was tested under damage degrees of 15%, 30%, and 50%. The content and particle size range of peanut shell powder were the same as in Section 3.1.3. The test was conducted at a temperature of 25 °C with a healing time of 10 min. The test results are shown in Figure 8. The HI of all asphalt samples significantly increased as the damage degree decreased. The maximum value was reached at a damage degree of 15% and a particle size range of 80–100 mesh, which was 90.9%. This is as expected, as larger damage degrees result in more microcracks within the asphalt, making it more difficult to recover. Greater damage degrees lead to more microcracks in the asphalt, making recovery challenging. Proactive, timely maintenance can prevent severe cracks, extending road service life and reducing costs.

### 3.2. Anti-Fatigue Properties of Peanut Shell Powder-Modified Asphalts

#### 3.2.1. Effect of the Peanut Shell Powder Content on the Fatigue Life and Fatigue Factor of Modified Asphalt

**Figure 9:**
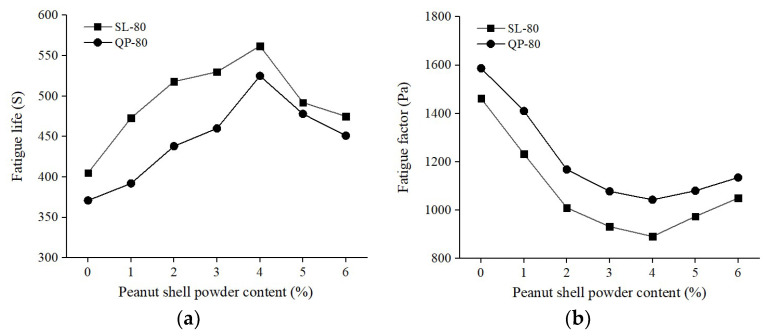
Effect of the peanut shell powder content on the anti-fatigue properties of modified asphalt. (**a**) Fatigue life; (**b**) Fatigue factor.

The fatigue life and fatigue factor of modified asphalt with different peanut shell powder contents were determined according to the test procedure described in Section 2.2.3. The particle size range of peanut shell powder was 80–100 mesh (180–150 μm). The test results are shown in Figure 9. The incorporation of peanut shell powder enhanced the fatigue life of the asphalt, and when the content of peanut shell powder was 4%, the modified Shuanglong asphalt exhibited the highest fatigue life, reaching 562 s, which was 38.8% higher than the base asphalt. After exceeding a 4% content, the rate of increase in fatigue life of the modified asphalt slowed down. This might be attributed to an excessive amount of peanut shell powder disrupting the uniformity and overall integrity of the modified asphalt, resulting in a reduction in fatigue life.

The fatigue factor reflected the shear modulus lost by the asphalt during the fatigue damage process. A smaller value indicated slower fatigue damage development and better fatigue resistance [35]. Similar to the fatigue life results, the fatigue factor initially decreased and then increased with increasing content of peanut shell powder. When the content was 4% and the base asphalt was Shuanglong asphalt, the fatigue factor was at its minimum value, indicative of optimal fatigue resistance. This phenomenon could be attributed to the fact that after exceeding the optimal value, the asphalt could not provide additional light oils due to the presence of peanut shell powder. The excess peanut shell powder led to a decrease in the overall uniformity of the modified asphalt, resulting in a rapid decline in fatigue resistance.

#### 3.2.2. Effect of the Particle Size of Peanut Shell Powder on the Fatigue Life and Fatigue Factor of Modified Asphalt

**Figure 10:**
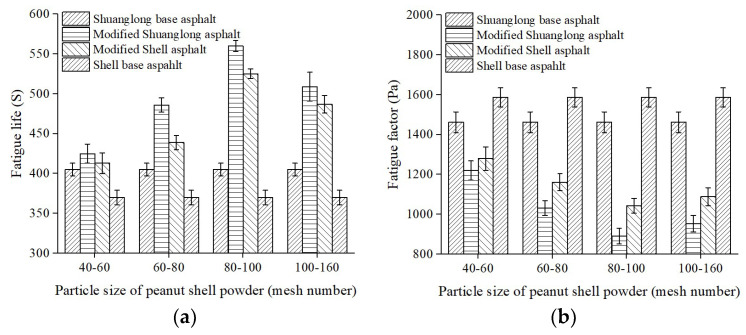
Effect of the particle size of peanut shell powder on the anti-fatigue properties of modified asphalt. (**a**) Fatigue life; (**b**) Fatigue factor.

In this fatigue test, the content of peanut shell powder was 4%, and other testing conditions were same to previous experiments. The test results are shown in Figure 10. Similar to the variation trend of the HI in Section 3.1.2, the fatigue life of the modified asphalt exhibited an initial extension followed by reduction with increasing particle size of peanut shell powder. When the particle size of the peanut shell powder ranged from 80 to 100 mesh, the modified Shuanglong asphalt exhibited a peak fatigue life of 562 s, marking a 38.8% increase over the base asphalt.

The fatigue factor initially decreased and then increased with increasing particle size of peanut shell powder. When the particle size was in the range of 80–100 mesh and the base asphalt was Shuanglong asphalt, the fatigue factor reached its minimum, indicating the best fatigue resistance. This suggests that when the particle size of peanut shell powder is too large, it has a smaller specific surface area and absorbs fewer light components. However, when the particle size of peanut shell powder exceeds 100 mesh, the volume of the particles becomes too small, resulting in insufficient void volume and inadequate absorption of light components, which is unfavorable for the fatigue resistance of the modified asphalt.

### 3.3. Conventional Properties of Peanut Shell Powder-Modified Asphalts

**Figure 11:**
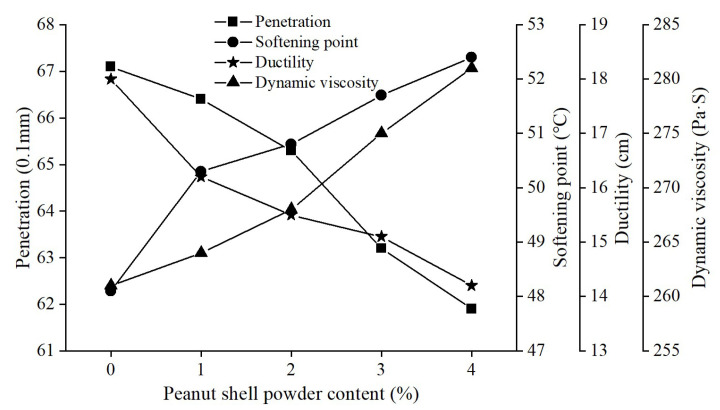
Effect of peanut shell powder content on the conventional properties of modified asphalt.

The penetration grading system for asphalt is the primary method employed in China to assess asphalt products. Corresponding tests were conducted to evaluate the impact of peanut shell powder on the conventional performance of asphalt. The penetration at 25 °C, softening point, ductility at 10 °C, and dynamic viscosity at 60 °C of peanut shell powder-modified asphalt were tested according to Section 2.2.4. Each test was repeated at least three times, and the valid average values were taken as the final results. Based on the results of the self-healing property test and fatigue property test, the optimal particle size range for peanut shell powder was determined to be 80–100 mesh, and the optimal content was 4%. Properties start to decline when the content exceeds this level. Therefore, modified asphalt samples were prepared with peanut shell powder content of 1%, 2%, 3%, and 4%, with a particle size range of 80–100 mesh. The test results were shown in Figure 11.

With the increasing content of peanut shell powder, the penetration and ductility of modified asphalt gradually decreased, while the softening point and dynamic viscosity gradually increased. When the content of peanut shell powder reached 4%, these indicators showed the greatest magnitude of change. The penetration and ductility decreased by 7.8% and 21.1%, respectively, while the softening point and dynamic viscosity increased by 8.9% and 7.1%, respectively. This was because the increased content of peanut shell powder absorbed more light components from the asphalt, resulting in increased viscosity and stiffness of the asphalt. This improved the high-temperature performance of the asphalt, but the decrease in ductility indicated a slight loss in low-temperature performance, which still remained within the range specified by standards [36].

**Figure 12:**
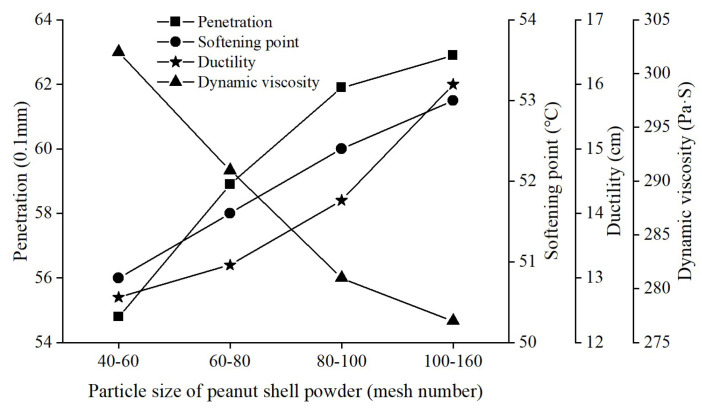
Effect of particle size of peanut shell powder on the conventional properties of modified asphalt.

The influence of peanut shell powder particle size on the conventional properties of modified asphalt was investigated, with a 4% peanut shell powder content. The results are shown in Figure 12. As the particle size of peanut shell powder decreased, the penetration, softening point, and ductility of the modified asphalt showed an increasing trend, while the dynamic viscosity gradually decreased. When the particle size range of peanut shell powder was 100–160 mesh, these indicators exhibited the greatest magnitude of change. The penetration, softening point, and ductility increased by 14.7%, 4.3%, and 25.9%, respectively, while the dynamic viscosity decreased by 8.3%. Overall, the changes were not significant, but a smaller particle size of peanut shell powder favored the self-healing performance of the modified asphalt.

### 3.4. Rheological Properties of Peanut Shell Powder-Modified Asphalts

**Figure 13:**
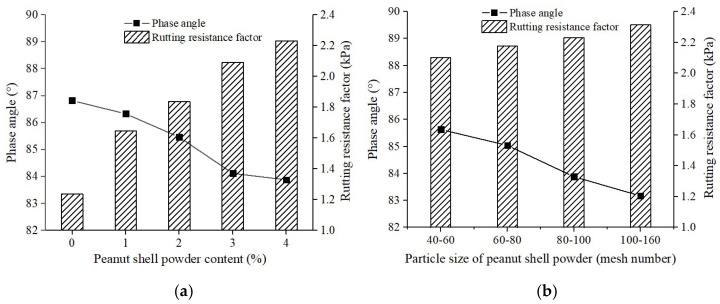
Effect of peanut shell powder on high temperature rheological property of asphalt. (**a**) Peanut shell powder content; (**b**) Particle size of peanut shell powder.

Figure 13 presented the influence of peanut shell powder content and particle size range on the high-temperature rheological properties of modified asphalt. The phase angle represented the ability of asphalt to recover from deformation after shear force was removed, with lower values indicating stronger recovery capability. The rutting factor represented the ability of asphalt to resist shear deformation at high temperatures [38]. Based on the reduction in phase angle and increase in rutting factor shown in Figure 13, it was concluded that increasing the content of peanut shell powder and reducing its particle size improved the high-temperature performance of asphalt, consistent with the results of the softening point test. This was likely due to the fact that peanut shell powder not only absorbed light oil fractions in asphalt but also played a reinforcing role due to its porous structure, enhancing the viscosity, stiffness, and overall stability of the asphalt.

**Figure 14:**
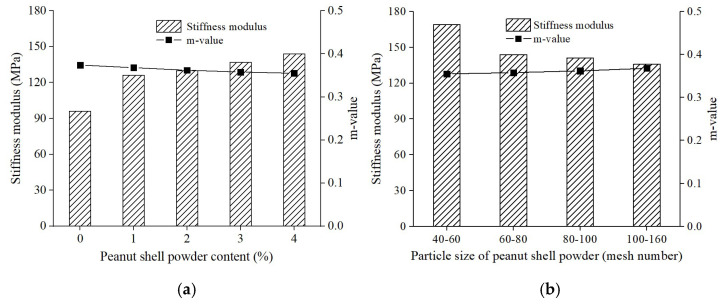
Effect of peanut shell powder on low temperature rheological property of asphalt. (**a**) Peanut shell powder content; (**b**) Particle size of peanut shell powder.

Figure 14 presented the influence of peanut shell powder content and particle size range on the low-temperature rheological properties of modified asphalt. According to findings from the Strategic Highway Research Program in the United States, the low-temperature rheological properties of asphalt are correlated with its high-temperature rheological properties, primarily reflecting the asphalt’s crack resistance under low-temperature conditions. The Stiffness modulus represents the deformation capacity of the modified asphalt, while the m-value represents the stress relaxation capacity. A smaller Stiffness modulus value and a larger m-value indicate better low-temperature cracking resistance of the modified asphalt [39]. Increasing the content of peanut shell powder raised the stiffness modulus of modified asphalt and decreased its m-value, which was detrimental to the crack resistance of modified asphalt at low temperatures, consistent with the results of the ductility test. At a peanut shell powder content of 4%, the stiffness modulus remained significantly below the required value of 300 MPa, while the m-value exceeded the specified threshold of 0.3 [36]. Alternatively, reducing the particle size of peanut shell powder lowered the stiffness modulus of modified asphalt and increased the m-value, thereby benefiting the low-temperature rheological properties of modified asphalt.

### 3.5. AFM Test of Peanut Shell Powder-Modified Asphalts

**Figure 15:**
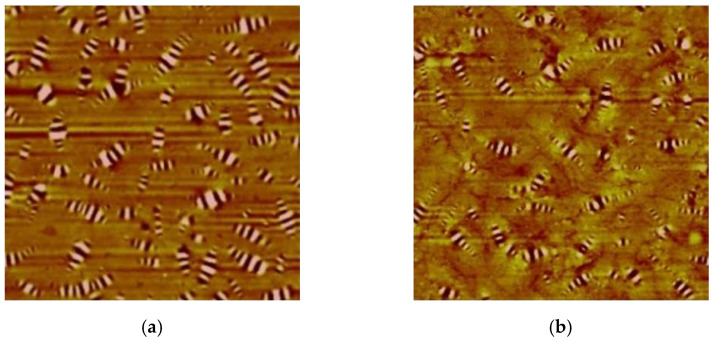

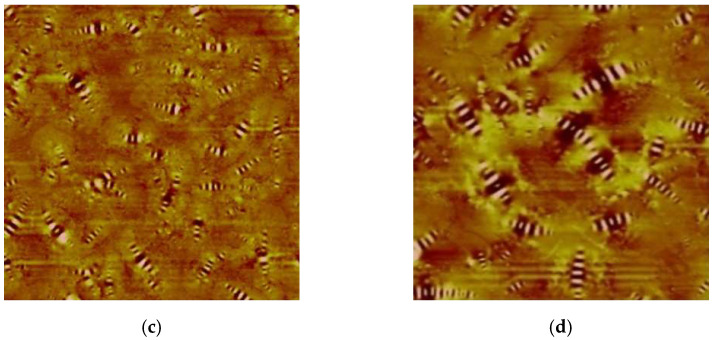
AFM 2D images of peanut shell powder-modified asphalt binders. (**a**) SL-80-0; (**b**) SL-80-2; (**c**) SL-80-4; (**d**) SL-80-6.

**Figure 16:**
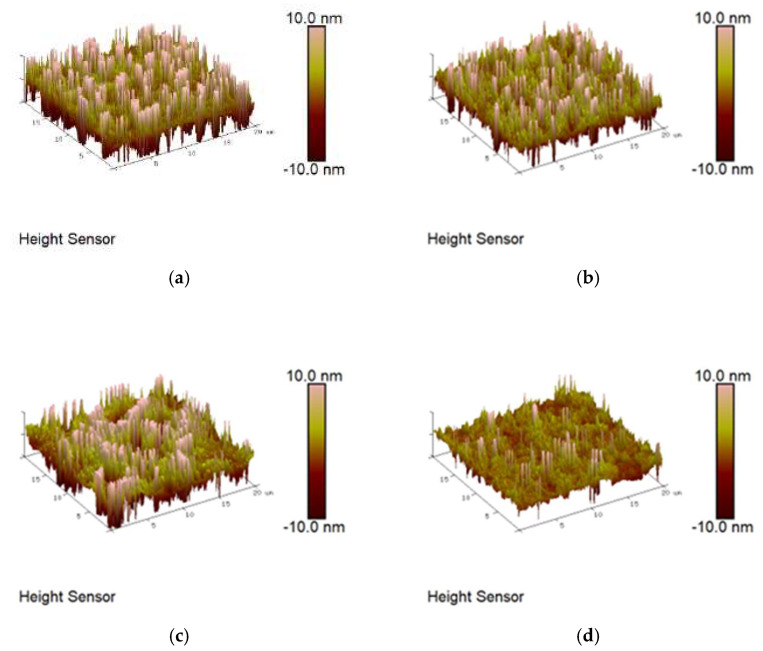
AFM 3D images of peanut shell powder-modified asphalt binders. (**a**) SL-80-0; (**b**) SL-80-2; (**c**) SL-80-4; (**d**) SL-80-6.

Atomic force microscopy (AFM) was used to scan the microstructure of modified asphalt samples with peanut shell powder particle sizes of 80–100 mesh and content levels of 0%, 2%, 4%, and 6%. The 2D and 3D micrographs of the modified asphalt were depicted in Figure 15 and Figure 16, respectively. The asphalt surface exhibits alternating black and white regions with different areas and sizes, which are named “bee-like structures”. In the 3D topography image, the “bee-like structure” appears as columnar structures with varying depths and bright white color [40]. Yang et al. [41] discovered through their research that there is a competitive relationship between asphaltene, wax, and light components within the asphalt. The “bee-like structure” mainly reflects the redistribution of asphaltene, wax, and other heavy components in the asphalt. Therefore, the morphological changes arising from the “bee-like structure” confirm the modification mechanism of asphalt by peanut shell powder at a microscopic level. The porous structure of peanut shell powder absorbs the light components within the asphalt, leading to an increased relative proportion of heavy components and consequently resulting in a more distinct “bee-like structure”.

With the addition of peanut shell powder, the quantity of “bee-like structure” per unit area in the asphalt gradually decreases with increasing peanut shell powder content, and some “bee-like structures” exhibit a slight increase in length. This could be attributed to the absorption of certain light components in the asphalt by the peanut shell powder, resulting in an increased relative proportion of asphaltene and wax and causing asphaltene aggregation. From the 3D images, the “bee-like structures” in the base asphalt appear as peaks, and with an increased content of peanut shell powder, these structures become more pronounced and significantly taller, while the surrounding morphology gradually decreases in height and tends to become smoother. Liu et al. [42] suggested that the roughness of asphalt’s microstructure is negatively correlated with its high-temperature performance, and the smoother microstructure of the peanut shell powder-modified asphalt compared to the base asphalt indicates its superior high-temperature performance, consistent with results of macroscopic performance tests.

**Figure 17:**
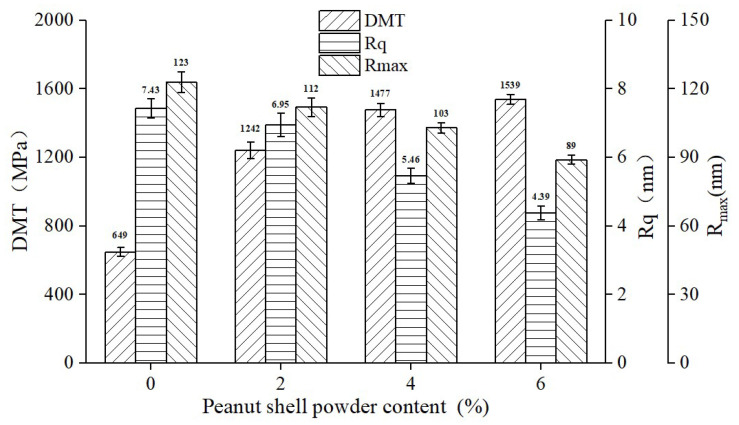
AFM factors of the peanut shell powder-modified asphalt.

The roughness factors R_q_ and R_max_ of the asphalt microsurface, as well as the DMT modulus, were obtained through AFM, as shown in Figure 17. The addition of peanut shell powder resulted in a decreasing trend in both R_q_ and R_max_, indicating a reduction in the roughness of the asphalt microsurface and a smoother microsurface, consistent with the results from the 2D and 3D topography images. Additionally, the incorporation of peanut shell powder led to an increase in the DMT modulus of the modified asphalt. The overall reduction in microsurface roughness of the asphalt is due to peanut shell powder absorbing the light components within the asphalt, leading to a decrease in the number of “bee-like structures”. Additionally, these light components are relatively softer constituents within the asphalt, resulting in an elevated DMT modulus of the asphalt. The DMT modulus reflects the asphalt’s resistance to deformation at the microscopic level, which aligns with the trend of macroscopic test indicators such as softening point and complex shear modulus variations. This consistency indicates an improvement in the high-temperature performance of the modified asphalt.

## 4. Conclusions

This study investigated the feasibility of improving the self-healing performance of asphalt using peanut shell powder. Modified asphalts with different peanut shell powder particle size ranges and contents were prepared, and the improvement in self-healing performance was evaluated using a fatigue–healing–fatigue test. The study also examined the effects of other factors such as healing time, damage degree, and test temperature on the performance of peanut shell powder-modified asphalt. Additionally, tests and analyses were conducted on the conventional properties, rheological properties, and microsurface morphology of the modified asphalt. The following conclusions are drawn:

(1) The content and particle size range of peanut shell powder had a significant impact on the improvement of self-healing performance in modified asphalt. The enhancement of self-healing performance in peanut shell powder-modified Shuanglong asphalt was higher than that in Shell asphalt. When the content of peanut shell powder was 4% and the particle size was 80–100 mesh, the modified asphalt showed a 79.4% increase in the self-healing index compared to the base asphalt. Correspondingly, the peanut shell powder-modified asphalt exhibited improved fatigue resistance;
(2) Experimental conditions or external environmental factors influenced the self-healing performance of asphalt. Increasing the ambient temperature enhanced the flow properties of asphalt molecules, while longer healing durations heightened the effectiveness of this movement, benefiting the restoration of asphalt performance. However, excessive fatigue damage reduced the self-healing capability of asphalt, emphasizing the importance of timely maintenance;
(3) Increasing the content of peanut shell powder and reducing its particle size improved the penetration, softening point, and viscosity of the modified asphalt. This indicated that peanut shell powder increased the stiffness of the asphalt and reduced its temperature sensitivity, which was crucial for maintaining excellent workability of the asphalt at high temperatures. However, they had a negative impact on the low-temperature ductility;
(4) The addition of peanut shell powder increased the rutting resistance factor of the asphalt and enhanced its rheological properties under high-temperature conditions. However, it also elevated the stiffness modulus of the asphalt and reduced its creep rate, which was detrimental to the asphalt’s resistance to deformation ability under low-temperature conditions;
(5) Atomic force microscopy results revealed a reduction in the roughness of the microstructure and an enhancement in the DMT modulus for peanut shell powder-modified asphalt. This improvement benefits the high-temperature performance of the asphalt and aligns with the macroscopic performance test results;
(6) This investigation offers a new perspective for enhancing the research and application of asphalt’s self-healing capabilities. Utilizing porous plant waste as a self-healing agent not only addresses the complexities and high costs associated with current self-healing agent preparation methods but also elevates the sustainability of waste utilization. To address the deficiency in low-temperature crack resistance of peanut shell powder-modified asphalt, further research can be conducted, followed by performance validation through asphalt mixture testing on the road.

## Figures and Tables

**Table 1:** Properties of peanut shell powder.

Measurements	Actual Value
Density/(g/cm^3^)	0.7
Melting point/°C	330
Flash point/°C	239.5

**Table 2:** The properties of Shuanglong and Shell base asphalt.

Measurements	Shuanglong	Shell
Penetration (25 °C)/0.1 mm	67.1	63.2
Softening point/°C	48.1	48.6
Ductility (10 °C)/cm	18.0	25.5
Dynamic viscosity (60 °C)/Pa·S	261	282
Mass loss/%	0.5	0.4
Performance Grade	64–22	64–22

**Table 3:** The labeling principles for modified asphalt.

Content of Peanut Shell Powder/%	Particle Size of Peanut Shell Powder/Mesh	Shuanglong Base Asphalt	Shell Base Asphalt
1	40–60	SL-40-1	QP-40-1
2	60–80	SL-60-2	QP-60-2
3	80–100	SL-80-3	QP-80-3
4	100–160	SL-100-4	QP-100-4

## Data Availability

Not applicable.
